# Recombinant human diamine oxidase prevents histamine-induced hypoxia, shock and death in guinea pigs

**DOI:** 10.1007/s00011-026-02297-4

**Published:** 2026-06-20

**Authors:** Felix Kosta, Matthias Weiss-Tessbach, Elisabeth Gludovacz, Birgit Reiter, Martin Murauer, Bernd Jilma, Marlene Rager-Resch

**Affiliations:** 1 Department of Clinical Pharmacology, Medical University of Vienna, Waehringer Guertel 18-20, 1090 Vienna, Austria; 2 Department of Biotechnology and Food Science, BOKU University, Muthgasse 18, 1190 Vienna, Austria; 3 Analytical Toxicology, Department of Laboratory Medicine/Joint Metabolome Facility, Medical University of Vienna, Waehringer Guertel 18-20, 1090 Vienna, Austria

**Keywords:** Histamine, Anaphylaxis, Diamine oxidase, Adrenaline, Enzyme therapy

## Abstract

**Background and objective:**

Histamine is the principal effector of anaphylaxis, and circulating levels correlate well with symptom severity in Hymenoptera venom–induced anaphylaxis. Adrenaline, the recommended first-line therapy, does not neutralize histamine and may cause relevant adverse effects. Recombinant human diamine oxidase (rhDAO), engineered with a mutated heparin-binding motif, exhibits improved pharmacokinetics and rapid histamine-degrading activity. The primary aim of this study was to evaluate whether prophylactic or particularly therapeutic rhDAO prevents or reduces histamine-induced shock compared with adrenaline in guinea pigs.

**Methods:**

Guinea pigs received subcutaneous histamine (1 mg/kg) with or without intramuscular adrenaline or intravenous rhDAO (2–8 mg/kg). Mean arterial pressure, heart rate, and arterial oxygen partial pressure (PaO₂) were monitored. Plasma histamine and rhDAO levels were measured.

**Results:**

Histamine injection raised plasma levels above 200 ng/ml and consistently induced shock with 42% mortality. Prophylactic and therapeutic rhDAO completely degraded circulating histamine, reduced shock incidence from 100% to 0% at the highest doses, shortened tachycardia, improved PaO₂ and decreased mortality. Intramuscular adrenaline (0.005 or 0.01 mg/kg) did not improve hemodynamics, oxygenation or survival.

**Conclusion:**

Recombinant hDAO prevented and treated histamine-induced shock and hypoxia, whereas adrenaline was ineffective. These findings support clinical development of rhDAO for life-threatening histamine-mediated conditions, including anaphylaxis.

**Supplementary Information:**

The online version contains supplementary material available at 10.1007/s00011-026-02297-4.

## Introduction

Histamine is the principal mediator of anaphylaxis [1]. Plasma levels of histamine can increase 100-fold during severe anaphylaxis [1–4]. During an acute allergic reaction, histamine is released from degranulating mast cells and basophils. However, as basophils contain only about 1% of the body’s total 100 mg histamine pool, mast cells serve as the primary source [1, 5]. Tissue-resident mast cells abruptly release their stored histamine into the vicinity, resulting in a rapid and substantial increase in tissue histamine concentrations [6]. Histamine overflow into the circulation triggers systemic effects regardless of the degranulation site [7, 8]. Normal plasma concentrations of histamine are below 1 ng/ml [9] but severe hypotension can already occur at > 3–5 ng/ml [10]. Histamine levels can reach several 100 ng/ml in severe anaphylaxis [2–4]. In very severe reactions to contrast media or Hymenoptera venom (6–12%), plasma histamine levels can exceed 400 ng/ml and may reach up to 1000 ng/ml [2, 4]. Increasing plasma histamine correlates with symptom aggravation, ranging from mild like flush and headache to life-threatening hypotension, bronchoconstriction and cardiac arrest [3, 11, 12]. There is a tight correlation between plasma histamine levels and the degree of hypotension or shock [4, 10].

Histamine activates four different receptors (H1R, H2R, H3R, H4R), of which H1R and H2R are mainly responsible for the induction of vasodilation [13, 14] and vascular leakage [12], both involved in shock development . In human histamine challenge experiments H1 and H2 blockers had limited effect, allowing only a 4-fold increase in tolerated histamine compared with controls [11]. 

Human anaphylaxis guidelines recommend early intramuscular injections of adrenaline with doses ranging from 0.005 to 0.01 mg/kg, with a single maximal dose of 0.5 mg [15], but no randomized clinical trials confirm its efficacy in anaphylaxis due to ethical and practical limits [16]. Although repetitive adrenaline injections are recommended in human anaphylaxis [17], adrenaline increased the death rate of protracted anaphylactic shock in guinea pigs dose dependently [18]. A trial in anaphylactic dogs did not indicate efficacy of intramuscular adrenaline [19]. Adrenaline has a narrow therapeutic window with many potential complications [20, 21]. 

There is a need for a safer and more effective treatment approach directly targeting the primary cause of severe hypotension during anaphylaxis. Diamine oxidase (DAO) is a phylogenetically highly conserved histamine-degrading enzyme [22]. In humans high DAO activity is measured in the gastrointestinal tract, the kidneys and the placenta [22–25]. Irreversible inhibition of DAO by aminoguanidine caused significant mortality during oral histamine challenge in pigs [26] and morbidity in sheep [27]. Pre-treatment of guinea pigs using DAO extracted from pea seedlings delayed time to death in anaphylaxis [28]. Gludovacz et al. produced recombinant human DAO (rhDAO) in a mammalian protein expression system [29]. Mutations of the rhDAO heparin-binding motif increased the alpha half-life from < 4 min to 6 h in rats, which could translate to several days in humans [30, 31]. 

In human anaphylaxis an important therapeutic goal would be to prevent progression from initial cardiovascular signs such as tachycardia to severe hypotension and cardiovascular collapse. Prophylactic rhDAO prevented tachycardia in guinea pigs during a 60 min constant intravenous histamine infusion (8 µg/kg/min) [32]. A more stringent objective is to reverse a state of shock, where tachycardia is accompanied by hypotension. As guinea pigs did not develop overt shock, hypoxia or mortality during continuous intravenous histamine infusion [32], the model was adapted to a subcutaneous bolus dose of 1 mg/kg histamine, which invariably induces shock in guinea pigs. We hypothesized that both prophylactic and, more importantly, therapeutic rhDAO infusions would lower shock rates compared to standard intramuscular adrenaline therapy. 

## Materials and methods

### Histamine-induced shock model

All experiments were conducted at the Medical University of Vienna following Austrian national animal research regulations under license #2020 − 0.193.044.

Animal procedures were performed on female Dunkin Hartley guinea pigs (380–600 g) housed at the Core Facility Laboratory Animal Breeding and Husbandry (Vienna, Austria). To meet welfare requirements for group housing, and to avoid the need for castrating and strictly supervising males, we restricted the study to females. Previous DAO dosing in other rodent species has revealed no sex-specific effects [30, 33, 34]. Sex is not an associated risk factor for contrast media-induced anaphylaxis [2]. The association between male sex and fatal neuromuscular-blocking-agent-induced anaphylaxis likely reflects confounding by cardiovascular comorbidity rather than a direct sex-related causal effect [35]. Following a minimum two-week acclimatization period, 7–8 week-old animals were used in the experiments. A comprehensive list of equipment and reagent suppliers is provided in the supplementary methods.

Anesthesia was induced subcutaneously with a mixture of medetomidine (0.1 mg/kg), midazolam (1 mg/kg), fentanyl (0.03 mg/kg) and ketamine (10 mg/kg). Animals were placed on a 37 °C heating pad and monitored for body temperature via a rectal probe.

Oxygen (0.7 l/min) was delivered via nose cone, and oxygen saturation was monitored using a pulse oximeter attached to the hind limb. A double-lumen catheter was placed into the jugular vein for intravenous infusion of etomidate (0.2 mg/kg/min) and fentanyl (0.03 mg/kg/h).

An arterial catheter was inserted into the carotid artery for invasive blood pressure monitoring, blood sampling and drug administration. Heart rate was measured using needle electrodes or via the arterial pressure waveform. Mean arterial pressure (MAP), heart rate and body temperature were recorded every minute.

Histamine base (320 µg/ml in 0.9% sterile sodium chloride (NaCl)) was injected subcutaneously into the left shoulder region at 1 mg/kg (3 ml/kg), because half (0.5 mg/kg) of the histamine dose did not induce shock in three animals. Control animals received 0.9% NaCl at the same volume.

Recombinant hDAO at 2, 4 or 8 mg/kg was infused via the carotid artery either directly after the first blood draw at minute 0 (prophylactically) or therapeutically after histamine injection.

Adrenaline diluted in 0.9% sterile NaCl was administered intramuscularly into the right or left thigh muscle at either 0.005 mg/kg or 0.01 mg/kg.

The time point for both therapeutic rhDAO and adrenaline administration was defined when the heart rate exceeded 250 beats per minute (bpm) for at least 1 min.

Partial arterial oxygen pressure (PaO_2_) was measured at the beginning and at the end of the experiment with the blood gas analyser ABL800-Flex.

Two blood samples of 270 µl and 180 µl were drawn every 10 min into a syringe pre-filled with either 30 µl 3.2% citrate or 20 µl 3.2% citrate + 500 µM diminazene aceturate. Diminazene aceturate is a potent and specific DAO inhibitor [36] used to prevent ex-vivo histamine degradation. The blood volume loss was immediately compensated by injection of 450 µl 0.9% NaCl containing 100 ng/ml Actilyse to maintain catheter patency.

After the 60-minute observation time all surviving animals were euthanized with 500 mg/kg pentobarbital administered via a venous or arterial catheter.

### Definition of shock

Shock was defined as a concurrent increase in heart rate and a drop in MAP. Animals were considered in shock when the heart rate exceeded the baseline value at minute 10 by ≥ 3 standard deviations (SD) and the MAP fell ≥ 30% below baseline for at least 5 consecutive minutes. Across all animals (*n* = 74) the mean (SD) baseline heart rate at minute 10 was 207 (15) bpm, establishing a heart rate threshold of 251 bpm or 1.21-fold baseline for shock.

### Definition of recovery from tachycardia

Recovery from tachycardia was defined as a return to the baseline heart rate at minute 10 plus 2 SDs (14.2%) for ≥ 5 consecutive minutes within 50 min after histamine injection, provided the animal survived the observation period.

### Definition of death time point

Death was defined either as a heart rate or a MAP of zero or a MAP < 10 mmHg with a heart rate < 100 bpm.

### Determination of histamine in plasma by liquid chromatography—tandem mass spectrometry

Histamine and ¹³C¹⁵N-histamine standards were quantified in plasma by a validated LC-MS/MS assay (Supplementary Methods), with method performance meeting ICH M10 criteria (Tables S1–S3).

### Diamine oxidase quantification and activity

The protein concentrations of rhDAO in guinea pig plasma were measured in duplicate with an enzyme-linked immunosorbent assay (ELISA) developed in-house [37] as previously described [32]. The activity of antibody-bound rhDAO was measured in duplicate with a H_2_O_2_ dependent Amplex Red assay as recently described [38]. 

### Statistical analysis

The significances in Kaplan-Meier plots were calculated using a pairwise Log-rank test with Bonferroni correction comparing each group individually to the control group (1 mg/kg histamine). The primary endpoint was time to shock. Mortality was a secondary endpoint. For mortality analysis, prophylactic (*n* = 18) and therapeutic (*n* = 24) groups were pooled and compared with the histamine group (*n* = 12). PaO₂ values and histamine concentration curve AUCs were analysed by one-way analysis of variance (ANOVA) with Tukey’s multiple comparison.

Five of 12 (42%) histamine-only, 6 of 10 (60%) adrenaline 0.005 mg/kg and 6 of 10 (60%) adrenaline 0.01 mg/kg animals died before blood sampling. Their missing values were replaced with the median of survivors of the respective group. As this underestimates the severity of symptoms in the control groups, it induces a conservative bias against active treatment which improves survival rates.

One animal from the 4 mg/kg therapeutic group died 11 min after histamine injection. This case is included in the respective group in Fig. 2, shown individually in Fig. 3A and D, Fig. S1 and Fig. S2, but excluded from statistical analysis in Fig. 3E.

## Results

### Recombinant human diamine oxidase but not adrenaline reduces histamine-induced shock and mortality

**Fig. 1:**
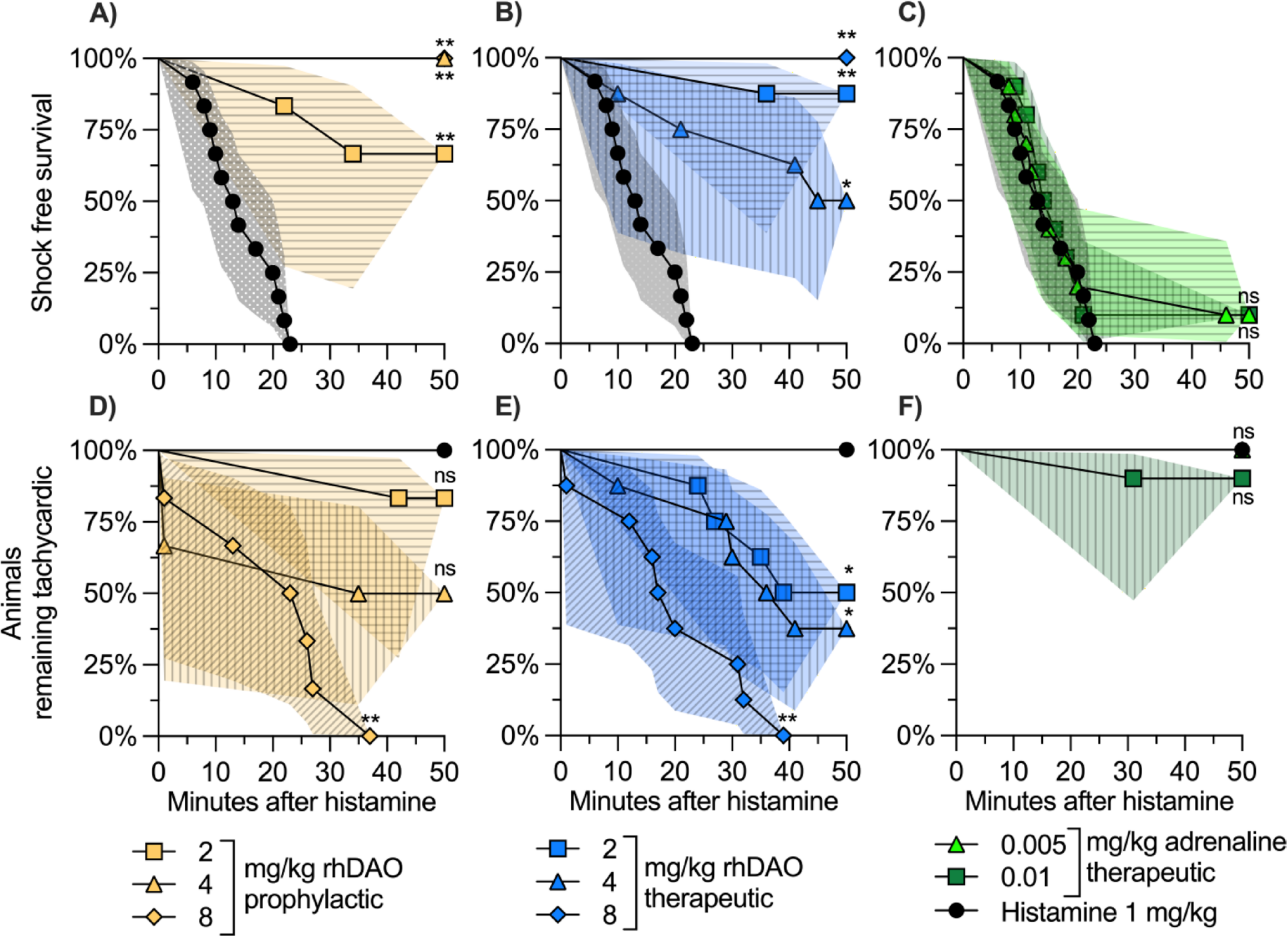
Recombinant hDAO but not adrenaline improves shock-free survival rates and accelerates recovery from histamine-induced tachycardia. **A**–**C** Kaplan–Meier curves showing the probability of remaining shock-free following subcutaneous histamine injections (1 mg/kg). **A** Histamine-only (n = 12) vs. prophylactic rhDAO (2, 4, 8 mg/kg; n = 6 each). **B** Therapeutic rhDAO groups (2, 4, 8 mg/kg; n = 8 each) **C** Adrenaline-treated groups (0.005, 0.01 mg/kg; n = 10 each). **D**–**F** Kaplan–Meier curves showing the reciprocal probability of recovery from histamine-induced tachycardia. **D** Histamine-only (n = 12) vs. prophylactic rhDAO (2, 4, 8 mg/kg; n = 6 each). **E** Therapeutic rhDAO groups (2, 4, 8 mg/kg; n = 8 each). **F** Adrenaline-treated groups (0.005, 0.01 mg/kg; n = 10 each). Therapeutic rhDAO or adrenaline was administered after tachycardia onset at a mean (SD) of 7.3 (1.9) and 7.4 (2.3) minutes after histamine injection respectively. Statistical comparisons were performed against the histamine-only group (Panels A–C; D–F) using the log-rank test with Bonferroni correction (ns *P* > 0.00625; *P < 0.00625; **P < 0.000625). Shaded areas represent 95% confidence intervals.

**Fig. 2:**
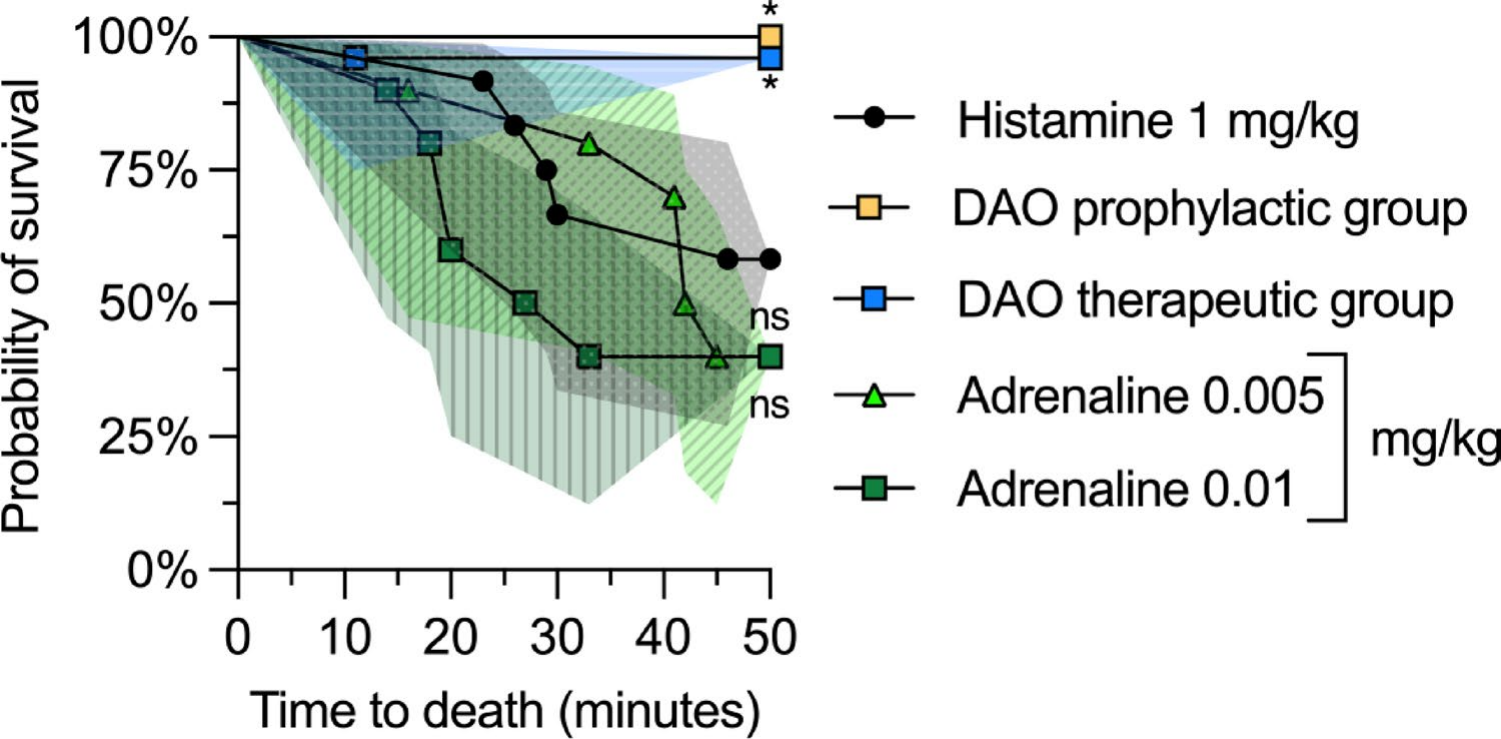
Recombinant hDAO but not adrenaline reduces histamine-induced mortality. Kaplan–Meier curves of survival after subcutaneous histamine injection (1 mg/kg at minute 0). Five out of twelve animals in the histamine-only group died (at minute 23, 26, 29, 30, and 46). No animals in the prophylactic rhDAO group (2, 4, and 8 mg/kg; n = 18) died. Out of 24 animals in the therapeutic rhDAO group, one animal (4 mg/kg) died at minute 11. In both adrenaline treatment groups six out of ten animals died (0.005 mg/kg at minute 16, 33, 41, 42, 42, and 45; 0.01 mg/kg at minute 14, 18, 20, 20, 27 and 33). Differences versus the histamine-only group were assessed by log-rank test with Bonferroni correction. ns *P* > 0.0125; *P ≤ 0.0125 Shaded areas represent 95% confidence intervals.

The bolus subcutaneous injection of 1 mg/kg histamine reliably induced shock within 23 min in all guinea pigs receiving just histamine (Fig. 1A–C). The average (SD) time to shock was 14.5 (5.9) minutes. None of these animals recovered from shock during the observation period. Prophylactic (Fig. 1A) or therapeutic (Fig. 1B) administration of the highest dose (8 mg/kg) of rhDAO protected all guinea pigs from histamine-induced shock. The 4 mg/kg prophylactic rhDAO dose also protected all guinea pigs from shock (Fig. 1A). Lower doses (2 mg/kg prophylactic, and 2 or 4 mg/kg therapeutic) significantly delayed shock onset and protected more than 50% of animals (Fig. 1A, B). Adrenaline in both doses (0.005 and 0.01 mg/kg) did not prevent or reduce shock in 90% of animals (Fig. 1C). Although shock occurrence was the predefined primary endpoint, mortality was assessed as a secondary outcome. Five of twelve animals (42%) died within 50 min in the histamine only group (Fig. 2). In contrast, no animals in the rhDAO prophylactic groups (0/18) and only one animal (1/24; 4%) in the rhDAO therapeutic groups died (Fig. 2). Adrenaline compared to histamine-only did not reduce mortality within 50 min after histamine showing a death rate of 60% in both adrenaline dose groups.

### Recombinant human diamine oxidase but not adrenaline reverses histamine-induced tachycardia

All animals receiving 1 mg/kg histamine developed persistent tachycardia (Fig. 1D–F). Tachycardia onset occurred on average (SD) at 7.5 (2.1, *n* = 56) minutes after injection. The mean (SD) time point of therapeutic rhDAO administration or adrenaline injection was 7.3 (1.9, *n* = 24) or 7.4 (2.3, *n* = 20) minutes respectively after injection of histamine. At a dose of 8 mg/kg rhDAO reversed tachycardia in all animals with heart rate normalizing between 37 and 39 min (Fig. 1D, E). 63% of animals receiving 4 mg/kg rhDAO therapeutically and 50% receiving 4 mg/kg prophylactically or 2 mg/kg therapeutically, recovered from tachycardia within 41, 35 and 39 min respectively (Fig. 1D, E). One of six animals (17%) in the 2 mg/kg prophylactic group returned to the baseline heart rate within 42 min (Fig. 1D). Adrenaline at 0.005 mg/kg failed to reverse tachycardia in all animals (Fig. 1F), while at 0.01 mg/kg it reversed tachycardia in 1 of 10 (10%) animals within 31 min (Fig. 1F).

### Recombinant human diamine oxidase but not adrenaline preserves oxygenation

**Fig. 3:**
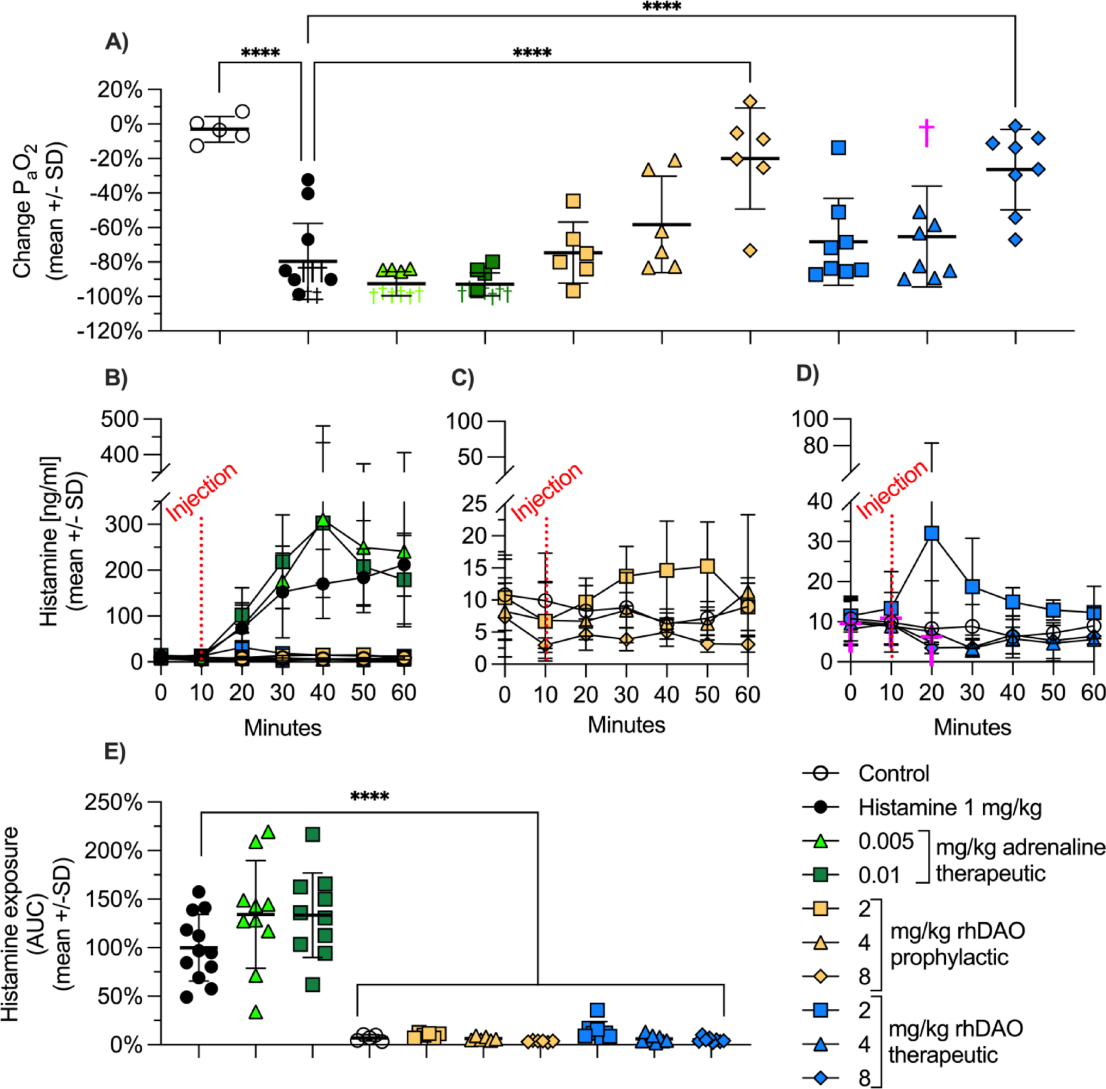
Recombinant hDAO but not adrenaline restores arterial oxygenation, reduces plasma histamine levels. **A** Percent change in partial arterial oxygen pressure (PaO₂) in individual animals measured 50 min after histamine injection or at time of death. **B** Plasma histamine concentrations (0–500 ng/ml) from experiment start through 60 min after histamine challenge. Groups shown in A and B include: NaCl control (n = 5), histamine-only (n = 12), prophylactic rhDAO (2, 4, 8 mg/kg; n = 6 each), therapeutic rhDAO (2, 4, 8 mg/kg; n = 8 each), and adrenaline-treated animals (0.005 and 0.01 mg/kg; n = 10 each). Histamine (1 mg/kg, s.c.) was administered at minute 10 (red dashed line in B–D). Therapeutic rhDAO or adrenaline was administered after tachycardia onset at a mean (SD) of 7.3 (1.9) and 7.4 (2.3) minutes after histamine injection, respectively. **C**, **D** Expanded views (0–100 ng/ml) of plasma histamine concentrations in prophylactic (**C**) and therapeutic (**D**) rhDAO groups. One non-surviving animal in the therapeutic 4 mg/kg group with death at 20 min is marked separately in panel D (†, magenta). **E** Percent changes in area under the curve (AUC) of plasma histamine concentrations from the time of histamine injection (minute 10) to end of the experiment (minute 60), relative to the mean of the histamine-only group. Groups were identical to those shown in panels A and B. For animals dying before 60 min, missing histamine values were replaced with the median of surviving animals in the same group. One animal in the 4 mg/kg therapeutic rhDAO group dying at 20 min is not shown in panel E. Animals dying before the end of the experiment are marked with † in panel A. Data represent mean ± SD in panels A–E, with individual values shown in panels A and E. Statistical analysis in panels A and E was performed using one-way ANOVA with Tukey’s multiple comparison test. ****P < 0.0001.

Histamine-induced shock severely impaired pulmonary oxygen exchange leading to systemic hypoxia. Fifty minutes after histamine injections, PaO₂ dropped by an average (SD) of 80% (22%) in the histamine-only group (Fig. 3A). Recombinant hDAO significantly mitigated this hypoxia with a mean (SD) PaO₂ decrease by only 20% (29%) in the 8 mg/kg prophylactic group and 26% (23%) in the therapeutic group. Adrenaline at both tested doses failed to improve oxygenation with both groups showing a marked mean (SD) decrease in PaO₂ of 93% (7%). This mean difference of 13% was not statistically significant compared with the histamine-only group.

### Recombinant human diamine oxidase reduces systemic histamine exposure

In the control group receiving no histamine, mean (SD) baseline plasma histamine concentrations were 9 (4) ng/ml throughout the experiment. Subcutaneous injection of 1 mg/kg histamine increased peak plasma levels to 211 (76) ng/ml (Fig. 3B). 50% of the mean maximal plasma concentration was reached after 29 (11) min. The area under the curve (AUC) reflects the total histamine exposure of the vasculature and was significantly reduced (*P* < 0.0001) by all prophylactic and therapeutic rhDAO doses (Fig. 3E). The 4 and 8 mg/kg rhDAO doses reduced the AUC by > 99% compared to only histamine administration. The AUC was reduced by 90% and 85% in the 2 mg/kg prophylactic and therapeutic group respectively. A temporary histamine increase occurred in the lowest therapeutic rhDAO dose group (Fig. 3D). The mean systemic histamine exposure of all rhDAO doses did not significantly differ (*P* > 0.9999) from the control group receiving saline (Fig. 3E). Adrenaline in both dose groups (0.005 and 0.01 mg/kg) did not reduce histamine exposure (Fig. 3B and E).

### The therapeutic intervention with recombinant human DAO acts rapidly

The median elevated heart rate plateaued 10 min after histamine injection (Fig. S1) and remained elevated throughout the observation period. Recombinant hDAO injection blunted the increase in heart rate within minutes (Fig. S1). The median drop of MAP values after 1 mg/kg histamine alone was most pronounced within the first 15 min (Fig. S1B). The drop in MAP was reversed by rhDAO within 3 min (Fig. S1B).

### Pharmacokinetics of recombinant human diamine oxidase

**Fig. 4:**
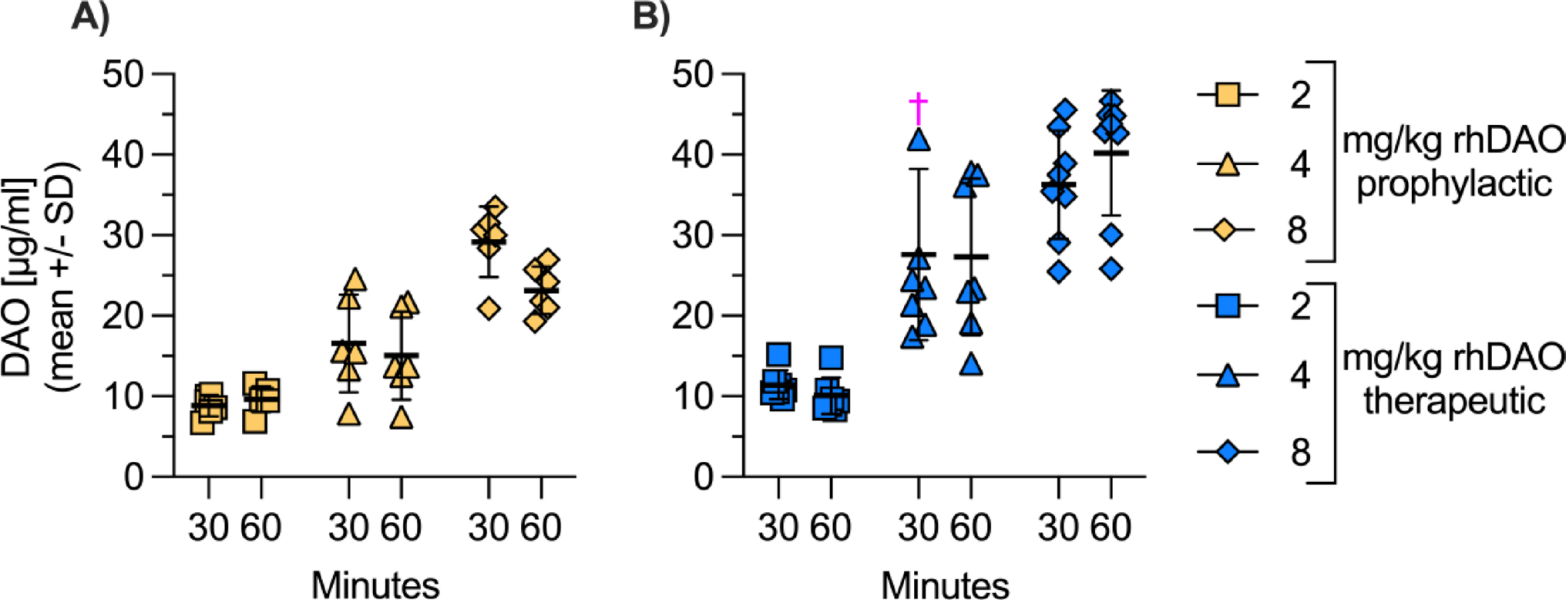
Recombinant hDAO achieves sustained systemic exposure. Plasma concentrations of recombinant human DAO (µg/ml) following **A** prophylactic and **B** therapeutic administration. Data represent mean ± SD.

Plasma concentrations of rhDAO increased dose-proportionally in the therapeutic groups and somewhat less in the prophylactic groups with stable levels from 30 to 60 min (Fig. 4A, B). The enzyme was fully active at all plasma concentrations for the entire observation period (Fig. S2).

## Discussion

Symptoms of anaphylaxis develop rapidly and the severity of anaphylaxis correlates with histamine plasma levels [2, 4]. Histamine released by mast cells is the cardinal mediator of anaphylactic shock in humans [1–4]. In humans intravenously infused histamine instantly and dose-dependently raises heart rate [11, 12] with hypotension and shock developing at > 2 ng/ml and 5 ng/ml, respectively [10]. In the most severe cases of anaphylaxis histamine concentrations can reach more than 200 ng/ml [2–4]. The guinea pig model used in the current investigations enabled assessment of rescue treatment under conditions of severe histamine-mediated cardiovascular collapse and direct comparison with intramuscular adrenaline.

In guinea pigs we used a 1 mg/kg subcutaneous bolus to mimic the acute histamine surge in human anaphylaxis. Continuous intravenous infusion of 8 µg/kg/min for 30 min caused only tachycardia, but not shock [32]. The current subcutaneous dose consistently induced shock and severe hypoxia. As tachycardia is the first cardiovascular sign of anaphylaxis in guinea pigs [32] onset of tachycardia prompted rhDAO infusion in the therapeutic group.

The 1 mg/kg subcutaneous histamine injection raised plasma histamine by > 50 ng/ml within 10 min, peaking above 200 ng/ml. This caused shock in all histamine-only animals, with none of the 7 survivors recovering from shock (Figs. 1A–C and 2). Observed shock percentages matched a published guinea pig bolus intravenous histamine challenge model [39], but the death rates were only 42% of prior values. Despite a dramatic drop in partial oxygen pressure (Fig. 3A) consistent with systemic hypoxia, animals did not suffocate, possibly due to oxygen insufflation or administration route.

Histamine concentrations of > 200 ng/ml are observed in the most extreme cases of severe anaphylaxis induced by contrast media [3], Hymenoptera stings [4] or in mastocytosis patients [40]. Such high histamine levels overwhelm antihistamines because in humans H1R and H2R blockers protect only when plasma histamine stays below 6 ng/ml [11, 41, 42]. Subcutaneous injection of a total histamine dose of 1 mg yielded peak plasma concentrations of 4.2 ng/ml in humans [43]. The increase in plasma histamine levels to 212 ng/ml in guinea pigs, which received a 60- to 80-fold higher histamine dose of 1 mg/kg compared to the 1 mg in humans, appears dose linear across species. The prolonged increase of plasma histamine in guinea pigs can be explained either by a protracted resorption of histamine from the subcutaneous tissue or by delayed excretion/metabolism during hypotension in guinea pigs. As histamine alone is sufficient to explain the degree of hypotension seen in human anaphylaxis [4], reducing the systemic histamine exposure is essential for hemodynamic stabilisation.

In guinea pigs all doses of rhDAO in both treatment regimens reduced the systemic histamine exposure by at least 85%. All guinea pigs receiving 8 mg/kg and those receiving 4 mg/kg rhDAO prophylactically were protected from shock and recovered from tachycardia (Fig. 1A, D). Therapeutic rhDAO acted immediately, halting or reversing the MAP decline (Fig. S1B). At 8 mg/kg therapeutic rhDAO briefly raised the MAP above baseline. This might be explained by ongoing and unopposed counter-regulatory catecholamine release lacking histamine as its vasodilatory counterpart.

The single intramuscular adrenaline treatment failed to improve shock, heart rate recovery, oxygenation or overall survival in histamine-challenged animals. While the intramuscular doses (0.005-0.0.1 mg/kg) used were clinically relevant [16, 44], they were associated with 60% mortality and with 90% development of shock in the guinea pig model. This aligns with previous animal and clinical studies, where adrenaline offered little hemodynamic benefit or caused cardiac side effects at higher doses including arrhythmia, ischemia and death [35, 45–47]. Previous studies in guinea pigs showed that in anaphylaxis adrenaline infusions increased lethality, especially at higher doses of 10 µg/min after intravenous administration [18, 48]. Studies in dogs also demonstrated that intramuscular adrenaline had no beneficial effects during anaphylaxis [19, 49]. Consistent with our findings, a recent randomized placebo-controlled human histamine challenge study demonstrated limited efficacy of intramuscular adrenaline in reversing histamine-induced hypotension [10]. Human case reports support limited efficacy, with repeated intramuscular injections failing to resolve symptoms [50, 51] or causing adverse outcomes after intravenous bolus delivery [46].

Recombinant hDAO rapidly reduced plasma histamine concentrations, the key mediator driving pathophysiological responses during human anaphylaxis [1] and consequently improved outcomes across all measured endpoints in our model. DAO-mediated histamine degradation generates hydrogen peroxide, ammonia and imidazole acetaldehyde as physiological metabolites. However, their expected levels remain low relative to endogenous background production and metabolic capacities. See supplement for further information.

Although all doses of rhDAO reduced shock rates, prophylactic administration of rhDAO appeared more effective in preventing shock occurrence. This is expected, because prophylactic rhDAO can degrade histamine immediately after release into the circulation. In contrast, histamine was able to exert its adverse effects for several minutes before rhDAO was infused in the therapeutic setting.

Doses of 2–8 mg/kg rhDAO showed dose-dependent plasma concentrations ranging from 10 to 40 µg/ml (Fig. 4A, B), far exceeding circulating DAO concentrations in healthy non-pregnant humans typically in the low ng/ml range, and even markedly elevated endogenous DAO concentrations reported during severe anaphylaxis in mastocytosis patients [23, 37, 52]. Overall higher concentrations are observed in the therapeutic group, which is likely due to the reported 20 min distribution phase [32] and the later time point of injection compared to the prophylactic setting. The K_M_ of rhDAO for histamine is 2.8 µM [22, 30], and 125 ng/ml DAO degraded 100 ng/ml (0.9 µM) histamine within 10 min in vitro [23]. In mice a plasma concentration of 10 µg/ml rhDAO reduced circulating histamine levels from > 700 to < 100 ng/ml [33]. In guinea pigs similar DAO plasma concentrations after 2 mg/kg rhDAO administration reduced histamine levels from ~ 200 ng/ml to ~ 10 ng/ml. Nevertheless, the lower doses of administered rhDAO may not be able to completely degrade the rapidly released histamine around the subcutaneous injection site, where the histamine concentration might be excessive. Guinea pigs are very sensitive towards pulmonary histamine, and small excess amounts of circulating histamine might impair lung function [53, 54]. This could possibly explain the lack of effect on PaO_2_ of the 2 and 4 mg/kg doses, compared with the 8 mg/kg rhDAO dose.

As the activity of rhDAO in plasma is very likely independent of species, it is conceivable that doses even lower than 2 mg/kg rhDAO can protect most humans from anaphylactic shock. Doses of 8 mg/kg rhDAO fully protected guinea pigs from shock caused by histamine concentrations of > 200 ng/ml. Therefore in humans it can be expected that lower doses of rhDAO of 0.5-1 mg/kg should suffice to rapidly degrade the median histamine concentrations of 13 ng/ml observed in anaphylactic shock after insect sting challenge [4]. A recent randomized placebo-controlled human histamine challenge study demonstrated that median histamine plasma levels of approximately 13 ng/ml were sufficient to induce a mean arterial pressure reduction of about 40 mmHg, comparable to Grade 4 Hymenoptera venom-induced anaphylaxis [4, 10]. Therefore, histamine appears sufficient and no other mediators are required to explain the degree of hypotension after Hymenoptera sting anaphylaxis.

After subcutaneous histamine challenge of guinea pigs, tachycardia developed within minutes closely resembling the symptom onset of < 5 min in patients with contrast media allergy [3]. In anaphylaxis, histamine overflows into the plasma within minutes in guinea pigs [39] and humans [4, 23]. Endogenous histamine levels remain elevated for several hours in some cases of severe anaphylaxis in humans [3, 55]. In rats the half-life of rhDAO with a mutated heparin binding domain is six hours, which could translate to multiple days in humans [30]. This prolonged exposure could also protect against biphasic anaphylaxis events mostly occurring within 8 h after the initial allergen exposure [55–57] and protracted or multiphasic anaphylaxis events lasting longer than a day [58].

One limitation of this study is that the observation period of the experiments was not long enough to determine the long-term survival of guinea pigs. Another limitation is that this animal study was not sufficiently powered to demonstrate improved survival in the individual dose groups. This would formally require more than 20 animals per dose group, based on the observed mortality rates of 42% in the control group and 2% in the DAO treatment groups using an alpha error of 5% and power of 90%. Considering the principles of the 3Rs, replacement, reduction and refinement, such a large animal trial does not seem justifiable. Nonetheless, pooling of treatment and dose groups indicated a survival advantage for rhDAO (Fig. 2). Guinea pigs are particularly susceptible to detrimental outcomes during and after surgery. One animal in the 4 mg/kg therapeutic rhDAO group died. Compared with other animals, its heart rate increased very rapidly in response to histamine before the rescue medication was administered. A very rapid resorption of histamine is possible resulting in severe stress during anaesthesia.

In conclusion, the subcutaneous injection of 1 mg/kg histamine into guinea pigs produced histamine concentrations found in the most extreme cases of severe anaphylaxis in humans. This reliably induced shock in all challenged animals. The therapeutic trial set-up showed for the first time the beneficial effects of the first-in-class biopharmaceutical rhDAO on histamine-induced shock and death. The inability of intramuscular adrenaline to act directly on the key mediator histamine was reflected by the lack of improvement in vital parameters and death rates. The superior efficacy of rhDAO over adrenaline provides a strong rationale for a histamine-targeted therapy in moderate to severe anaphylaxis. These data allow the calculation of rhDAO doses for future human histamine challenge studies to investigate its potency to inactivate life-threatening histamine concentrations.

## Supplementary Information

Below is the link to the electronic supplementary material.

Supplementary Material 1

## Data Availability

Data available on request from the authors.

## Abbreviations

ANOVA: Analysis of variance
AUC: Area under the curve
DAO: Diamine oxidase
rhDAO: Recombinant human diamine oxidase
ELISA: Enzyme-linked immunosorbent assay
MAP: Mean arterial pressure
bpm: Beats per minute
PaO_2_: Partial arterial oxygen pressure
SD: Standard deviation
