# Supplementary material for: Recombinant human diamine oxidase prevents histamine-induced hypoxia, shock and death in guinea pigs
Source: Inflamm Res. 2026 Jun 20;75(1):146. doi: 10.1007/s00011-026-02297-4 (PMC13283141; doi:10.1007/s00011-026-02297-4)
Supplement: Supplementary file 1 — Supplementary Material 1 [file 11_2026_2297_MOESM1_ESM.docx]

**Supporting information to:**

**Recombinant human diamine oxidase prevents histamine-induced hypoxia, shock and death in guinea pigs.**

Felix Kosta MSc^1^, Matthias Weiss-Tessbach MD, PhD^1^, Elisabeth Gludovacz PhD^2^, Birgit Reiter PhD^3^, Martin Murauer MD^1^, Bernd Jilma MD^1^, Marlene Rager-Resch DVM^1^*

^1^Department of Clinical Pharmacology, Medical University of Vienna, Waehringer Guertel 18-20, 1090 Vienna, Austria

^2^Department of Biotechnology and Food Science, BOKU University, Muthgasse 18, 1190 Vienna, Austria

^3^Analytical Toxicology, Department of Laboratory Medicine/Joint Metabolome Facility, Medical University of Vienna, Waehringer Guertel 18-20, 1090 Vienna, Austria

*Correspondence:

Marlene Rager-Resch DVM, Department of Clinical Pharmacology, Medical University of Vienna, Waehringer Guertel 18-20, Vienna, Austria

Email: marlene.rager-resch@meduniwien.ac.at


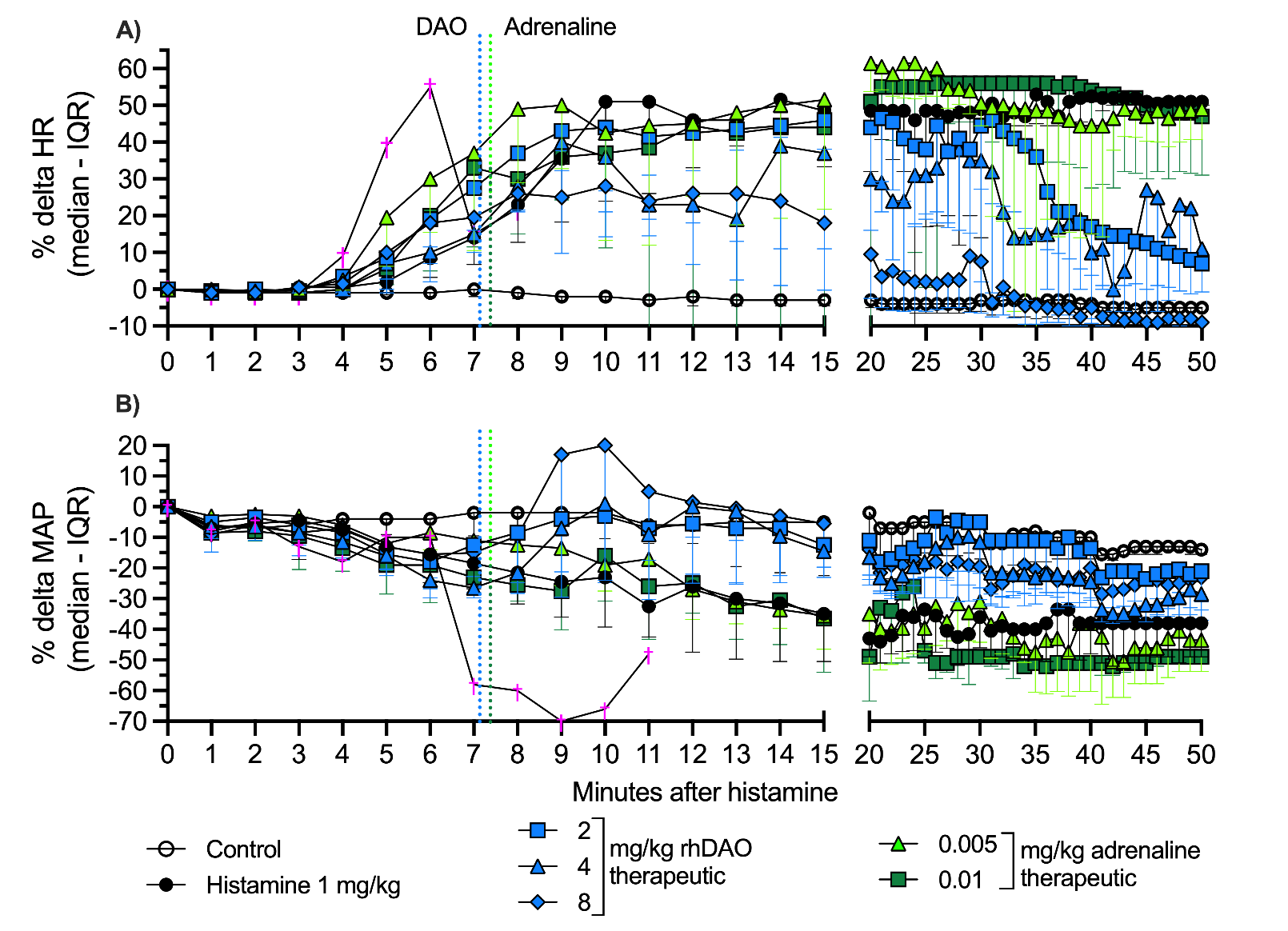
**Figure S1: Recombinant hDAO but not adrenaline restores hemodynamic stability after histamine challenge.**

Legend figure S1: **A, B)** Median percent change (minus interquartile range, IQR) in **A)** heart rate (HR) and **B)** mean arterial pressure (MAP), relative to the time of histamine injection. Groups include: NaCl control (n = 5), histamine-only (n = 12), therapeutic rhDAO (2, 4, and 8 mg/kg; n = 8 each), and adrenaline-treated animals (0.005 and 0.01 mg/kg; n = 10 each). Mean injection times of rhDAO and adrenaline are indicated by blue and green vertical lines respectively.


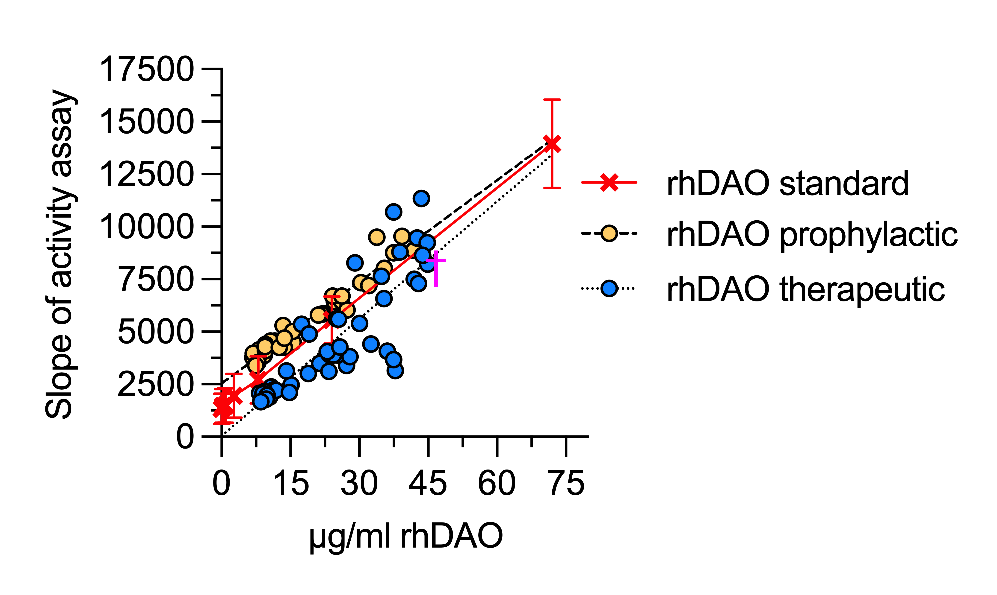
**Figure S2: Recombinant hDAO was fully active at all plasma concentrations.**

Legend figure S2: Recombinant hDAO was spiked into guinea pig plasma to generate a standard curve (0–72 µg/ml) and showing a strong linear correlation between ELISA-measured concentrations and Amplex Red–based enzymatic activity (slope = 174 per µg/ml, R² = 0.95). Data from 30- and 60-minute plasma samples of all rhDAO-treated guinea pigs over all doses are shown for prophylactic (slope = 163, R² = 0.95; n = 6 per dose) and therapeutic (slope  = 187, R² = 0.71; n = 8 per dose) groups. One animal in the 4 mg/kg therapeutic group dying at 20 minutes is marked separately (†, magenta).

**Supplementary methods**

**Reagents and Equipment**

| **Medetomidine** | Domitor; Provet, Switzerland |
| --- | --- |
| **Midazolam** | Midazolam Accord; Accord Healthcare, Austria |
| **Fentanyl** | Fentanyl Primal; Primal Critical Care, Netherlands |
| **Ketamine** | Ketanest; Pfizer, Austria |
| **Double lumen catheter** | Nutri-line Twinflow, 2 Fr; Vygon, Germany |
| **Etomidate** | Etomidate-Lipuro; Braun, Germany |
| **Arterial catheter** | Umbilicalcath Expert, 2.5 Fr; Vygon, Germany |
| **0.9% sodium chloride** | Sigma-Aldrich, USA |
| **Adrenaline** | Suprarenin; Sanofi, Germany |
| **Blood Gas Analyzer ABL800Flex** | Drott, Austria |
| **Pentobarbital** | Release, WDT; Garbsen, Germany |

**Determination of histamine in plasma by liquid chromatography – tandem mass spectrometry (LC-MS/MS)**

Histamine dihydrochloride was purchased from Supelco. Radioactive ^13^C^15^N-histamine dihydrochloride was ordered from Sigma Aldrich. Standards were dissolved in water.

All samples were analyzed by an QTRAP 6500 + system equipped with a Turbo Ion Spray Source operated in positive electrospray ionization mode and an Exion AD LC system (both Sciex, Framingham, MA, USA) which was equipped with a Cortecs UPLC HILIC 1.6 µm column, 2.1 mm x 100 mm (Waters, Milford, MA, USA).

Gradient elution was performed using 100 mM ammonium formate adjusted to pH 3 with formic acid as mobile phase A and acetonitrile as mobile phase B at a flow rate of 0.55 ml/min and an oven temperature of 45°C. Quantification was performed by multiple reaction monitoring in positive mode with the following mass transitions: m/z 111.9 🡪 m/z 95.0 for histamine and m/z 113.9 🡪 96.0 for ^13^C^15^N-histamine.

Ten µl of sample was mixed with 10 µl internal standard (0.25 µg ^13^C^15^N-histamine/ml water), precipitated with 100 μl acetonitrile, vortexed for 5 sec and centrifuged at 20.800 x g and 4°C for five minutes. 5 µl of the supernatant were injected into the LC-MS/MS system. Method validation was performed according to International Council for Harmonisation guideline M10 on bioanalytical method validation (EMA/CHMP/ICH/172948/2019) considering the endogenous nature of the analyte.

Detailed data for accuracy and precision are listed in supplement tables S1-S3.

**Table S1-3:** **Detailed validation data for accuracy and precision of the histamine LC-MS/MS method.**

|  |  | **Within-day precision and accuracy (*n* = 5)** | | | **Between-day precision and accuracy (*n* = 9)** | | |
| --- | --- | --- | --- | --- | --- | --- | --- |
| **5% BSA** | **Nominal concentration (ng/ml)** | **Mean concentration (ng/ml)** | **RSD (%)** | **Bias (%)** | **Mean concentration (ng/ml)** | **RSD (%)** | **Bias (%)** |
| LLOQ | 2.5 | 2.7 | 3.3 | 8.0 | 2.6 | 8.6 | 5.8 |
| lQC | 7.5 | 6.8 | 2.0 | -9.6 | 7.2 | 5.9 | -4.2 |
| mQC | 75 | 70.5 | 2.7 | - 6.0 | 72.7 | 4.0 | -3.1 |
| hQC | 125 | 114.0 | 1.3 | - 8.9 | 124.8 | 6.1 | -0.2 |

**Table S1:** Within- and between-day precisions (expressed as relative standard deviations (RSD)) and accuracies (expressed as bias) for histamine controls (lower limit of quantification – quality control (LLOQ – QC), low QC (lQC), medium QC (mQC) and high QC (hQC)) prepared in 5% BSA PBS.

For the assessment of precision in authentic plasma samples the concentration of a plasma pool was determined by analysis against the surrogate matrix curve. This represents the unspiked lQC. Two further QCs were prepared by spiking this plasma with the results listed in Table S2.

|  |  | **Within-day precision and accuracy (*n* = 5)** | | **Between-day precision and accuracy (*n* = 9)** | |
| --- | --- | --- | --- | --- | --- |
| **Plasma**  **Level** | **Nominal concentration (ng/ml)** | **Mean concentration (ng/ml)** | **RSD (%)** | **Mean concentration (ng/ml)** | **RSD (%)** |
| lQC | 2.98 | 2.4 | 3.4 | 2.5 | 11.5 |
| mQC | 82.98 | 82.4 | 9.0 | 92.5 | 9.4 |
| hQC | 132.98 | 131.6 | 4.8 | 139.5 | 10.3 |

**Table S2:** Within- and between-day precisions (expressed as RSD) for histamine controls prepared in lithium heparin plasma (lQC = unspiked sample, mQC and hQC spiked sample)

The assessment of accuracy in plasma was performed by analyzing mQC and hQC ( = spiked plasma samples) and by calculation using the following formula (according to ICH M10 guideline):


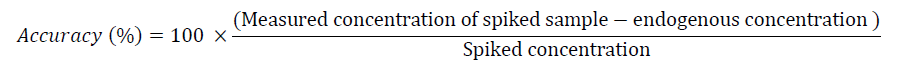


|  | **mQC spiked plasma** | |  | **hQC – spiked plasma** | |
| --- | --- | --- | --- | --- | --- |
|  | **Nominal concentration**  **83.0 ng/ml** | **Acc (%)** |  | **Nominal concentration**  **133.0 ng/ml** | **Acc (%)** |
| Sample Prep 1 | 68.7 | 82.1 |  | 140.6 | 105.9 |
| Sample Prep 2 | 86.0 | 103.8 |  | 128.1 | 96.2 |
| Sample Prep 3 | 81.9 | 98.6 |  | 137.8 | 103.7 |
| Sample Prep 4 | 90.8 | 109.8 |  | 125.6 | 94.3 |
| Sample Prep 5 | 84.5 | 101.9 |  | 125.8 | 94.5 |
| **Mean** | **82.4** | **99.2** |  | **131.6** | **98.9** |

**Table S3:** Accuracy (Acc) in % of spiked plasma mQC and hQC (fivefold sample preparation and single analysis).

**Research In Context**

**Evidence before this study**

Previous work demonstrated that prophylactic recombinant human diamine oxidase (rhDAO) attenuated histamine-mediated physiological effects in animal models. In DAO knockout mice, prophylactically administered rhDAO reduced histamine-induced temperature loss and clinical symptoms in wild-type C57BL6 mice, which are rather histamine resistant.[1] However, these experiments did not measure blood pressure drop, shock, hypoxia, mortality or other symptoms induced by histamine during cardiovascular collapse in human anaphylaxis or mast cell activation syndrome. Subsequently, prophylactic rhDAO attenuated haemodynamic instability during continuous intravenous histamine infusion in guinea pigs.[2] However, this model did not induce overt shock, hypoxia or mortality and therefore did not allow assessment of rescue treatment under conditions resembling severe histamine-mediated cardiovascular collapse. More recently, a randomised placebo-controlled human histamine challenge study demonstrated limited efficacy of intramuscular adrenaline in reversing histamine-induced hypotension in healthy volunteers.[3] However, for obvious safety reasons this human model could not test higher histamine exposure as encountered in severe anaphylaxis [4–6], nor effects on hypoxia or mortality. Additionally, rhDAO is currently not approved in humans and therefore could not be tested in this human shock model. No previous studies directly compared rhDAO with guideline-consistent intramuscular adrenaline treatment in severe histamine-mediated shock.

**Added value of this study**

The present study established a severe histamine-mediated shock model in guinea pigs using subcutaneous histamine bolus administration, reproducibly resulting in shock, hypoxia and substantial mortality. This enabled assessment not only of prophylactic but also therapeutic rhDAO administration after shock onset. Importantly, the present study also directly compared rhDAO with guideline-consistent intramuscular adrenaline dosages in severe histamine-mediated shock. Recombinant human diamine oxidase rapidly degraded circulating histamine, improved oxygenation, reduced shock rates and markedly improved survival. In contrast, clinically relevant intramuscular adrenaline doses did not significantly improve shock rates, oxygenation or survival. The study therefore demonstrates the efficacy of therapeutic histamine degradation during severe histamine-mediated cardiovascular collapse.

**Implications of all the available evidence**

Collectively, including previous research [7] the available evidence supports histamine as a central mediator of severe cardiovascular dysfunction during anaphylaxis and suggests that direct enzymatic histamine degradation may represent a mechanistically targeted therapeutic approach. These findings provide a rationale for further translational and clinical investigation of rhDAO for severe histamine-mediated conditions including anaphylaxis.

**Supplementary discussion**

**Safety considerations of metabolites formed during degradation of histamine by diamine oxidase in anaphylaxis**

**Hydrogen peroxide:**

In extreme cases of Hymenoptera venom anaphylaxis, plasma histamine levels reach peak concentrations of 1000 ng/mL (9 µM). Mean concentrations are approximately 1 µM in severe anaphylaxis, while median concentrations are 0.1 µM.[4] However, these high plasma histamine concentrations are short-lived. Since one molecule of H₂O₂ is formed by the oxidation of one molecule of histamine, recombinant human diamine oxidase (rhDAO) would release a median of 0.1 µM H₂O₂ into the circulation in cases of severe anaphylaxis.

For comparison, recombinant urate oxidase (rasburicase) decreases plasma uric acid from approximately 10 to 1 mg/dL (a non-linear decline of 9 mg/dL) within 4 hours.[8] This can be estimated to produce 400 µM H₂O₂ per hour within the vasculature. This 4000-fold higher H₂O₂ generation was still well tolerated, with negligible toxicity observed in recipients.

In general, the hydrogen peroxide-inactivating capacity of plasma is estimated to be 500 µM or more. We were only able to measure horseradish peroxidase/hydrogen peroxide coupling after consuming 200 µM DAO substrate.[9] Additionally, red blood cells, which contain catalases and peroxidases, increase the antioxidant capacity of plasma manifold. Hydrogen peroxide is both a substrate and a metabolite of many human enzymes. Therefore, we consider it unlikely that histamine-derived hydrogen peroxide causes toxic effects during severe mast cell activation or anaphylaxis.

**Ammonia:**

Diamine oxidase degrades histamine by oxidative deamination. Due to a 1:1 molar stoichiometry, the degradation of 1 mM (111 µg/ml) histamine produces 1 mM ammonia. Notably, 1 mM histamine represents a theoretical maximum, corresponding approximately to the total amount of histamine stored in the human body (Table E4 in [10]). In vitro stimulation with (anti-) IgE releases only about 25% of stored histamine from human mast cells.[11,12] Thus, in the worst-case scenario, 0.25 mM histamine would be released in the body, resulting in 0.25 mM NH₃. This is less than the background production of ~0.7 mM NH₃ per minute (daily amino acid turnover produces 1 M/70 kg/day NH₃.[13] Therefore, toxic effects due to NH₃ production by rhDAO can be ruled out.

**Imidazole Acetaldehyde:**

Imidazole acetaldehyde is rapidly converted to imidazole acetic acid, a metabolite with no described toxic effects. Despite extensive literature research, we found no references indicating that either metabolite is unsafe or toxic.

However, foods containing very high amounts of histamine, such as fermented soy products (200 mg/100 g), Roquefort cheese (200 mg/100 g), or balsamic vinegar (400 mg/100 g), are considered fairly safe. After consumption of such foods, the diamine oxidase in the gastrointestinal tract is expected to detoxify the histamine, producing similar or even higher amounts of these metabolites compared to severe anaphylaxis.

Based on limited published information, no adverse health effects were observed after exposure to 50 mg histamine for healthy individuals[14], whereas higher amounts may induce histamine mediated effects. Hence, no adverse effects from these histamine metabolites are expected when rhDAO is used to treat histamine excess in human anaphylaxis.

**Supplementary References**

1. Karer M, Rager-Resch M, Haider T, Petroczi K, Gludovacz E, Borth N, et al. Diamine oxidase knockout mice are not hypersensitive to orally or subcutaneously administered histamine. Inflamm Res. 2022;71:497–511. https://doi.org/10.1007/s00011-022-01558-2

2. Weiss-Tessbach M, Reiter B, Gludovacz E, Boehm T, Jilma B, Rager-Resch M. Recombinant human diamine oxidase prevents hemodynamic effects of continuous histamine infusion in guinea pigs. Inflamm Res. 2023;72:2013–22. https://doi.org/10.1007/s00011-023-01783-3

3. Weiss‐Tessbach M, Dizdarevic AM, Bischof T, Firbas C, Taschner A, Ritter‐Hobl E, et al. Effect of Intramuscular Adrenaline on Histamine‐Induced Hypotension: A Randomised Placebo‐Controlled Pilot Trial. Allergy. 2026;all.70277. https://doi.org/10.1111/all.70277

4. van der Linden PW, Hack CE, Poortman J, Vivié-Kipp YC, Struyvenberg A, van der Zwan JK. Insect-sting challenge in 138 patients: relation between clinical severity of anaphylaxis and mast cell activation. J Allergy Clin Immunol. 1992;90:110–8. https://doi.org/10.1016/s0091-6749(06)80017-5

5. Reitter M, Petitpain N, Latarche C, Cottin J, Massy N, Demoly P, et al. Fatal anaphylaxis with neuromuscular blocking agents: a risk factor and management analysis. Allergy. Wiley; 2014;69:954–9. https://doi.org/10.1111/all.12426

6. Clement O, Dewachter P, Mouton-Faivre C, Nevoret C, Guilloux L, Bloch Morot E, et al. Immediate Hypersensitivity to Contrast Agents: The French 5-year CIRTACI Study. EClinicalMedicine. 2018;1:51–61. https://doi.org/10.1016/j.eclinm.2018.07.002

7. Reber LL, Hernandez JD, Galli SJ. The pathophysiology of anaphylaxis. J Allergy Clin Immunol. 2017;140:335–48. https://doi.org/10.1016/j.jaci.2017.06.003

8. Pui CH, Mahmoud HH, Wiley JM, Woods GM, Leverger G, Camitta B, et al. Recombinant urate oxidase for the prophylaxis or treatment of hyperuricemia in patients With leukemia or lymphoma. J Clin Oncol Off J Am Soc Clin Oncol. 2001;19:697–704. https://doi.org/10.1200/JCO.2001.19.3.697

9. Boehm T, Karer M, Gludovacz E, Petroczi K, Resch M, Schuetzenberger K, et al. Simple, sensitive and specific quantification of diamine oxidase activity in complex matrices using newly discovered fluorophores derived from natural substrates. Inflamm Res. 2020;69:937–50. https://doi.org/10.1007/s00011-020-01359-5

10. Boehm T, Ristl R, Joseph S, Petroczi K, Klavins K, Valent P, et al. Metabolome and lipidome derangements during a severe mast cell activation event in a patient with indolent systemic mastocytosis. J Allergy Clin Immunol. 2021;148:1533–44. https://doi.org/10.1016/j.jaci.2021.03.043

11. He S-H, Xie H, He Y-S. Induction of tryptase and histamine release from human colon mast cells by IgE dependent or independent mechanisms. World J Gastroenterol. 2004;10:319. https://doi.org/10.3748/wjg.v10.i3.319

12. Dvorak A, Massey W, Warner J, Kissell S, Kagey-Sobotka A, Lichtenstein L. IgE-mediated anaphylactic degranulation of isolated human skin mast cells. Blood. 1991;77:569–78. https://doi.org/10.1182/blood.V77.3.569.569

13. Levitt D, Levitt M. A model of blood-ammonia homeostasis based on a quantitative analysis of nitrogen metabolism in the multiple organs involved in the production, catabolism, and excretion of ammonia in humans. Clin Exp Gastroenterol. 2018;Volume 11:193–215. https://doi.org/10.2147/CEG.S160921

14. Scientific Opinion on risk based control of biogenic amine formation in fermented foods. EFSA J. https://doi.org/10.2903/j.efsa.2011.2393
